# Complete Genome Sequence of a Peste des Petits Ruminants Virus Recovered from an Alpine Goat during an Outbreak in Morocco in 2008

**DOI:** 10.1128/genomeA.00096-13

**Published:** 2013-05-09

**Authors:** Murali Muniraju, Mehdi El Harrak, Jingyue Bao, Aravindh Babu Ramasamy Parthiban, Ashley C. Banyard, Carrie Batten, Satya Parida

**Affiliations:** The Pirbright Institute, Pirbright, Surrey, United Kingdoma;; Société de Productions Pharmaceutiques et Vétérinaires, Laboratoire de Virologie, Rabat, Moroccob;; China Animal Health and Epidemiology Center, Qingdao, Shandong, People’s Republic of Chinac;; Animal Health and Veterinary Laboratories Agency, Weybridge, Surrey, United Kingdomd

## Abstract

Here, we announce the first complete genome sequence of a field isolate of a peste des petits ruminants virus (PPRV) from northern Africa. This isolate is derived from an Alpine goat that suffered from severe clinical disease during the 2008 outbreak in Morocco. The full genome sequence of this isolate clusters phylogenetically with the lineage IV isolates of PPRV, sharing high levels of sequence identity with other lineage IV isolates.

## GENOME ANNOUNCEMENT

Peste des petits ruminants virus (PPRV) is a highly contagious viral pathogen of small ruminants (1) that is endemic across much of Africa, the Middle East, and Asia (2, 3). PPRV infection was considered to be an exotic disease limited to northern Africa until 2008, when an outbreak occurred in Morocco and the disease spread rapidly throughout the entire country between July and August (4). Goats were considered to be more susceptible than sheep to PPRV during this Moroccan outbreak, and alpine goats exhibited severe clinical disease, while local breeds of sheep developed milder clinical disease (5). The genome sequence presented here was derived from a mesenteric lymph node sample from an infected goat, following a single passage on Vero.DogSLAMtag cells.

As is typical of morbilliviruses, serologically, only one serotype of PPRV exists. However, following genetic characterization, PPRV isolates group into four genetically divergent lineages. Currently, full-genome sequence data are available for one virus isolate from lineage I, PPRV Côte d’ Ivoire/89, a highly virulent field isolate (GenBank accession no. EU267273); two virus isolates within lineage II, Nigeria/76/1 (accession no. EU267274), a mild field isolate from domesticated small ruminants, and Nigeria/75/1 (accession no. X74443), a tissue culture-passaged live-attenuated vaccine strain; and four pathogenic virus isolates from lineage IV, Turkey/2000 (accession no. NC_006383) and the Tibetan isolates Tibet/30/2007 (accession no. FJ905304), Tibet/2007 (accession no. JF939201), and Tibet/Bharal/2008 (accession no. JX217850). A further near-complete lineage IV genome sequence exists, that of the Indian vaccine strain Sungri/96, although the 3′ terminus remains undefined (accession no. AY560591). As previously demonstrated (2, 6), the Morocco/2008 isolate reported here belongs to lineage IV and is the first isolate derived from northern Africa to have the complete genome characterized.

Oligonucleotide primers were designed using the conserved regions of PPRV full-length genome sequences available in the database, as detailed above. The primers were used to generate seven overlapping amplicons of the Morocco/2008 isolate, which were gel purified and sequenced with an ABI-3730 automated sequencer (Applied Biosystems). The genome termini were determined using 3′/5′ rapid amplification of cDNA ends (RACE) (7). A total of 248 sequences were assembled into overlapping contigs that represented the full genome (Lasergene v.10.1), with an average of 6.7-fold coverage at each nucleotide position. The total genome size of Morocco/2008 is identical to those of PPRV isolates with previously published sequences, 15,948 nucleotides, with identical genome organization, including that in the regions encoding the nucleocapsid (N), phosphoprotein (P/C/V), and the matrix (M), fusion (F), hemagglutinin (H), and large polymerase (L) proteins. Genome and antigenome promoter regions, gene start and stop sequences, and intergenic trinucleotides were present, as expected. At the nucleotide level, Morocco/2008 shows 97.2% homology to strain Turkey/2000, 96.9% homology to strain Sungri/96, 97% homology to strains Tibet/30/07 and Tibet/07, 96.9% to strain Tibet/Bharal/2008, 92.5% and 92.6% to Nigeria/76/1 and Nigeria/75/1, respectively, and 89.5% homology to Côte d’ Ivoire/89. It is worth mentioning that the leader sequence of the Sungri/96 virus strain is not published and, therefore, it was not included in the nucleotide homology analysis.

### Nucleotide sequence accession number.

The full-genome sequence of PPRV Morocco/2008 is available in GenBank under the accession no. KC594074.
